# Enhanced focal cortical dysplasia detection in pediatric frontal lobe epilepsy with asymmetric radiomic and morphological features

**DOI:** 10.3389/fnins.2023.1289897

**Published:** 2023-11-15

**Authors:** Manli Zhang, Hao Yu, Gongpeng Cao, Jinguo Huang, Yanzhu Lu, Jing Zhang, Nana Liu, Wenjing Zhang, Yintao Cheng, Guixia Kang, Lixin Cai

**Affiliations:** ^1^Key Laboratory of Universal Wireless Communications, Ministry of Education, School of Information and Communication Engineering, Beijing University of Posts and Telecommunications, Beijing, China; ^2^Department of Pediatric Epilepsy Center, Peking University First Hospital, Beijing, China; ^3^School of Automation, Beijing University of Posts and Telecommunications, Beijing, China

**Keywords:** radiomics, morphological, FCD lesion localization, children, MRI, PET

## Abstract

**Objective:**

Focal cortical dysplasia (FCD) is the most common pathological cause for pediatric epilepsy, with frontal lobe epilepsy (FLE) being the most prevalent in the pediatric population. We attempted to utilize radiomic and morphological methods on MRI and PET to detect FCD in children with FLE.

**Methods:**

Thirty-seven children with FLE and 20 controls were included in the primary cohort, and a five-fold cross-validation was performed. In addition, we validated the performance in an independent site of 12 FLE children. A two-stage experiments including frontal lobe and subregions were employed to detect the lesion area of FCD, incorporating the asymmetric feature between the left and right hemispheres. Specifically, for the radiomics approach, we used gray matter (GM), white matter (WM), GM and WM, and the gray-white matter boundary regions of interest to extract features. Then, we employed a Multi-Layer Perceptron classifier to achieve FCD lesion localization based on both radiomic and morphological methods.

**Results:**

The Multi-Layer Perceptron model based on the asymmetric feature exhibited excellent performance both in the frontal lobe and subregions. In the primary cohort and independent site, the radiomics analysis with GM and WM asymmetric features had the highest sensitivity (89.2 and 91.7%) and AUC (98.9 and 99.3%) in frontal lobe. While in the subregions, the GM asymmetric features had the highest sensitivity (85.6 and 79.7%). Furthermore, relying on the highest sensitivity of GM and WM asymmetric features in frontal lobe, when integrated with the subregions results, our approach exhibited overlaps with GM asymmetric features (55.4 and 52.4%), as well as morphological asymmetric features (54.4 and 53.8%), both in the primary cohort and at the independent site.

**Significance:**

This study demonstrates that a two-stage design based on the asymmetry of radiomic and morphological features can improve FCD detection. Specifically, incorporating regions of interest for GM, WM, GM, and WM, and the gray-white matter boundary significantly enhances the localization capabilities for lesion detection within the radiomics approach.

## 1 Introduction

Frontal lobe epilepsy (FLE) is a common surgically treatable form of epilepsy, and it is more prevalent in children (Salanova et al., 1994; Laskowitz et al., 1995; Téllez-Zenteno et al., 2005). Malformation of cortical development (MCD), and in particular focal cortical dysplasia (FCD), is the most common pathological cause for pediatric epilepsy (Harvey et al., 2008; Lorio et al., 2021). Previous study demonstrates that preoperative diagnosis of lesions can improve postoperative outcomes (Téllez-Zenteno et al., 2010), so accurate detection and localization of epileptic lesions are crucial for resection plan and surgical efficacy.

A few studies have evaluated the cortical thickness (Widjaja et al., 2011; Rahatli et al., 2020) and cortical volume changes (Lawson et al., 2002; Rahatli et al., 2020) in children with FLE, these studies have found cortical changes in the frontal lobe compared to the control group. However, specific lesion localization studies are lacking. Currently, FCD lesion detection research has primarily focused on morphological abnormalities, such as cortical thickness, blurring at the gray-white matter boundary (GWM) and fluid-attenuated inversion recovery (FLAIR) signal intensity changes in MRI. Based on these morphological abnormalities, some studies employ surface-based features to identify focal abnormalities in a pediatric cohort (Adler et al., 2017; Kulaseharan et al., 2019) and Multi-centre Epilepsy Lesion Detection Project cohort (Spitzer et al., 2022). In order to highlight the cortical thickness and the blurred GWM feature of FCD, the Morphometric Analysis Program was employed for lesion localization (Huppertz et al., 2005; David et al., 2021). Additionally, PET imaging can assist in the localization of lesions by utilizing metabolic and asymmetric information (Mo J.-J. et al., 2019), which may aid the identification of occult FCD lesions that were missed on MRI. It has been demonstrated that the combination of MRI and PET has higher sensitivity compared to MRI alone (Tan et al., 2018).

In recent studies, radiomics approaches have shown promising performance in the directions of type 2 diabetes mellitus (Xu et al., 2022) and breast cancer (Huang et al., 2021), while also demonstrating excellent performance in the diagnosis of temporal lobe epilepsy (TLE) using MRI (Mo J. et al., 2019; Park et al., 2020; Cheong et al., 2021) and PET (Zhang et al., 2021) images. In radiomics method that focus on pediatric FCD research, a particularly relevant study employed a two-stage Bayes classifier (Kulaseharan et al., 2019). Initially, voxel classification was carried out based on cortical thickness and blurring at the GWM features. Subsequently, voxels classified as lesions were reclassified using texture features. In comparison to the utilization of first-order statistics and texture features in MRI (Mo J. et al., 2019), MRI and wavelet images (Cheong et al., 2021) were used for obtaining rich high-dimensional radiomics features in TLE. However, the application of radiomics either alone or in combination with morphological features in FCD research has not been extensively explored. Additionally, compared to the morphological method that primarily focuses on the region of interest (ROI) comprised of gray matter (GM), white matter (WM) and GWM, there is a lack of literatures available on the application of radiomics methods for analyzing these ROIs. Therefore, this study was conducted to investigate the radiomics method for identifying abnormalities in the GM, WM, and GWM.

Drawing from previous research, epilepsy has been conceptualized as a disruption at the level of the entire brain network (van Diessen et al., 2013; Wang et al., 2022). Additionally, earlier studies have found that in patients with unilateral frontal lobe epilepsy, compensatory mechanisms exist in the contralateral hemisphere (Swartz et al., 1996; Widjaja et al., 2014). Furthermore, FCD lesions in pediatric patients tend to be larger, and surgical interventions typically focus on resecting the epileptogenic zone rather than all abnormal areas. To enhance the accuracy of detecting epileptogenic zone and mitigate potential interference from contralateral regions in patients with unilateral FLE, We have designed a two-stage detection method from the frontal lobe to subregions. Moreover, this two-stage approach offers the advantage of a progressively refined detection process. In the first stage, we can more easily identify abnormalities within the cerebral hemisphere, thus narrowing down the search area for the lesion. Subsequently, in the second stage, we can concentrate on detecting abnormal regions within the affected frontal lobe, thereby enhancing the accuracy of FCD detection.

In this study, we employed a Multi-Layer Perceptro (MLP) network to analyze multimodal (including T1, FLAIR and PET) imaging data. We utilized radiomics methods to detect FCD by extracting features from different ROIs including GM, WM, GM and WM (GM&WM), and GWM, incorporating shape, first-order, and texture features from multimodal and wavelet images. Additionally, we incorporated asymmetric features from the left and right hemispheres. Based on the clinical diagnostic process of lesion localization, we devised a two-stage detection approach using radiomic and morphological approach. First, the presence of pathology was determined in the left and right frontal lobe regions. Subsequently, all subregions of the affected frontal lobe were discriminated to identify the specific areas of FCD. To ensure the generalizability of our method, we further validated the performance of our model on an independent site.

## 2 Materials and methods

### 2.1 Materials

#### 2.1.1 Participants

The FLE subjects we used for research were collected from the pediatric epilepsy center of Peking University First Hospital between 2016 and 2018, with the following inclusion criteria: (1) FLE in children; (2) Clinical suspicion of FCD or MCD; (3) The experimental data is a retrospective analysis study, so it is required to be seizure-free for at least one year after surgery. Since age and gender-matched healthy control groups of children including T1, FLAIR, and PET images are difficult to obtain, we used 20 children with TLE as the control group and underwent the same MRI and PET protocol from Peking University First Hospital between 2018 and 2021. In addition, other study also adapted patients with TLE as reference group (Tan et al., 2018). All patients in the control group underwent surgery and were histopathologically confirmed to have no FCD present. We conducted PET imaging confirmation to ensure the absence of metabolic abnormalities beyond the temporal lobe.

#### 2.1.2 MRI acquisition and data preprocessing

All participants contain 3-T T1, FLAIR and PET images. We obtained three-dimensional datasets using T1-weighted sequences (TR = 8.0 ms, TE = 3.8 ms, slice thickness = 1 mm, no gap, voxel size = 0.86 mm × 0.86 mm × 1 mm) and T2-FLAIR sequences (TR = 4,800 ms, TE = 277 ms, TI = 1,650 ms, FOV = 176 × 220 mm, slice thickness = 1 mm, no gap, voxel size = 0.98 mm × 0.98 mm × 0.56 mm). Simultaneously, we acquired brain PET images using 18F-FDG (slice thickness of 3 mm, voxel size of 2 mm) and performed attenuation correction using CT data.

**Figure 1:**
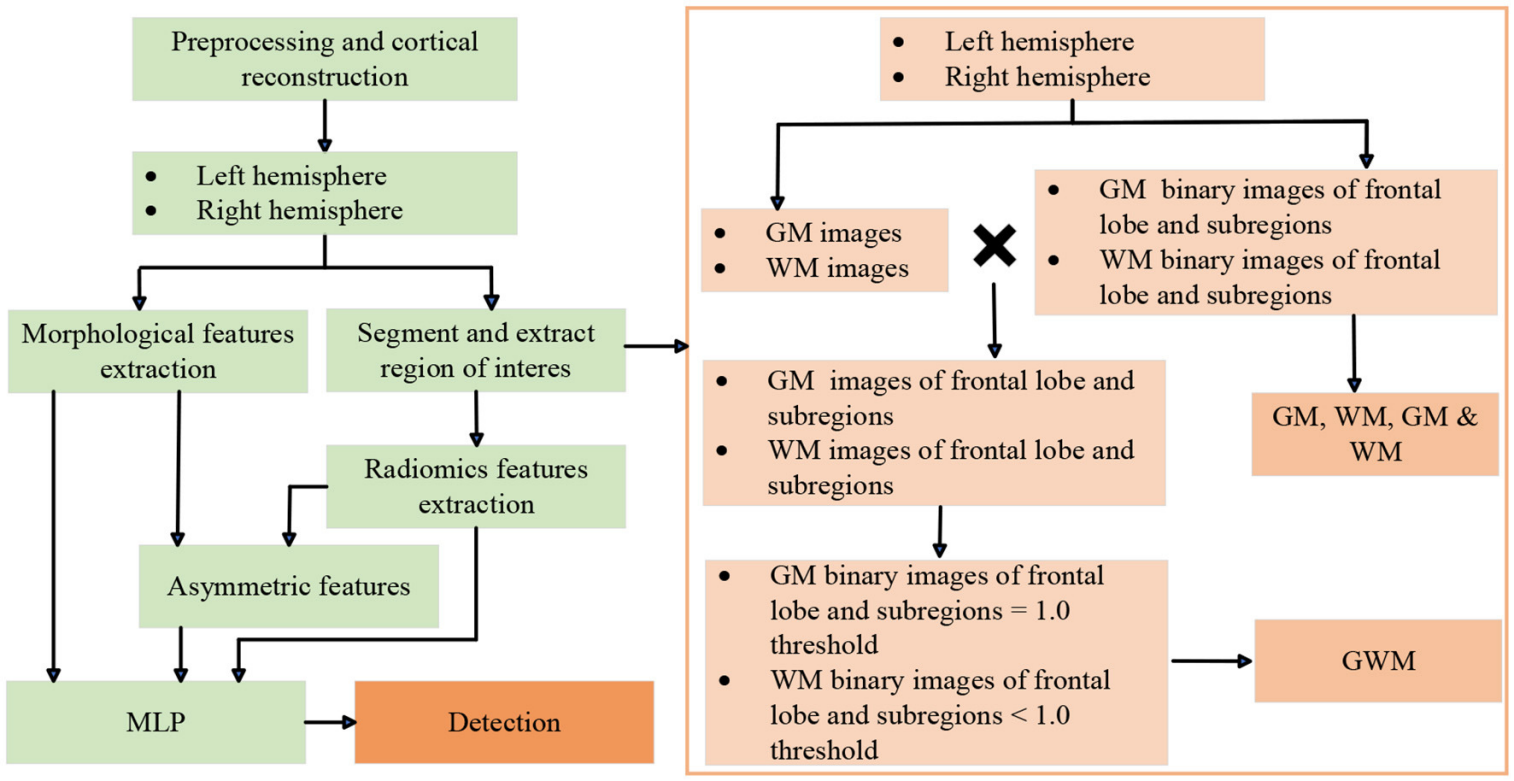
Flow dagram of experimental design. MLP, multi-layer perceptron. GM, gray matter; WM, white matter; GM & WM, GM and WM combined; GWM, boundary between GM and WM.

**Figure 2:**
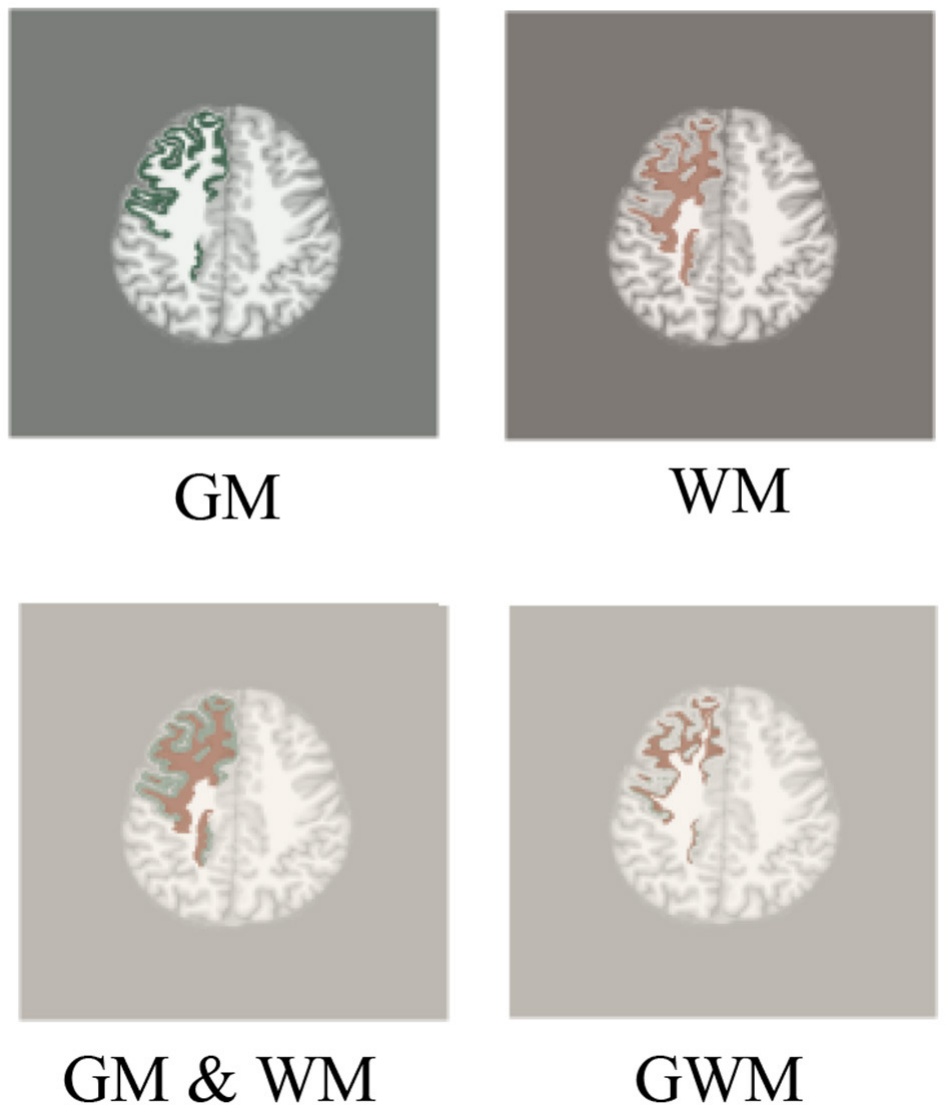
Region of interest of radiomics. We use T1 as background image to display region of interest, including GM, WM, GM & WM and GWM, similar to FLAIR and PET images.

The overview of the FCD processing pipeline is presented in Figures 1, 2, including ROIs segmentation and extraction steps, as well as visualizations. Further details are explained in the sections below. The first preprocessing step for all participants is to register the FLAIR and PET images to the T1 image through the Functional Magnetic Resonance Imaging of the Brain Software Library toolkit (Jenkinson et al., 2012), and cortical reconstruction using FreeSurfer v6 (Fischl, 2012). In brief, the T1 sequence was first resampled to achieve isotropic voxel sizes of 1 mm × 1 mm × 1 mm (Bossi Zanetti et al., 2023). Subsequently, head motion correction, intensity normalization, skull stripping, and white matter segmentation were performed. Following that, three-dimensional reconstruction was carried out to generate the reconstructed cortical brain. Lastly, the registered FLAIR sequence, PET data, and manual labels were mapped onto the reconstructed cortical brain. After preprocessing, Each FLE or control can be divided into two subjects, including left and right hemisphere. For each hemisphere, the frontal lobe and its subregions are the areas we need to analyze. Subregions are extracted from the Desikan-Killiany atlas (Desikan et al., 2006) and include: caudal middle frontal; frontal pole; lateral orbitofrontal; medial orbitofrontal; paracentral; pars opercularis; pars orbitalis; pars triangularis; precentral; rostral middle frontal; superior frontal. Specifically, the frontal lobe is the combination of all its subregions. The subsequent ROIs segmentation and feature extraction are performed separately on the frontal and subregions. For children with FLE, manual lesion labels were created based on postoperative resection areas, and the labels are then registered to the reconstructed cortical surface.

### 2.2 Methods

#### 2.2.1 Morphological/intensity features

All the features below are calculated in the individual space of all participants: (1) Cortical thickness; (2) Gray-white matter intensity contrast; (3) Mean curvaure; (4) Sulcal depth; (5) local cortical deformation (LCD); (6) FLAIR intensity; (7) Dougnnut method maps; (8) PET standardized uptake value ratios (SUVR); (9) GM volume; (10) WM volume. FLAIR intensity was sampled at the GWM and at depths of 25%, 50%, and 75% of cortical thickness, as well as at 0.5 and 1 mm below the GWM. The cortical thickness, gray-white matter intensity contrast, and FLAIR intensity were measured within the dougnnut method. Details about extraction of (1)–(7) features can be found elsewhere (Adler et al., 2017; Mo J.-J. et al., 2019; Spitzer et al., 2022). PetSurfer was used to calculate the PET SUVR (Greve et al., 2014, 2016). In order to reduce noise, the following features were smoothed with a 10 mm full-width-at-half-maximum gaussian kernel: cortical thickness; gray-white matter intensity contrast; LCD; FLAIR intensity; dougnnut method maps.

#### 2.2.2 Radiomic features

##### 2.2.2.1 Segment and extract ROIs

The steps of extracting various ROIs of frontal lobes and subregions can be seen in the Figure 1. Based on the reconstructed brains for all participants, the processing involved segmenting and extracting ROIs as follows: (1) GM and WM images were segmented using the SPM12 tool; (2) GM and WM binary images of frontal lobe and subregions were extracted from the Desikan-Killiany atlas; (3) ROIs of GM, WM, and GM & WM. (4) GM and WM images of frontal lobe and subregions; (5) GWM was obtained by screening the pixel values on the GM and WM images of the frontal lobe and subregions with thresholds of 1.0. At the boundary of the GM and WM images, the probability value of GM is relatively large, and the probability value of WM is relatively small. Therefore, the ROI of GM with a probability ≥1.0 is taken, and the ROI of WM with a probability < 1.0 is taken. All visualizations of the ROIs are shown in Figure 2.

##### 2.2.2.2 Radiomic features extraction

Based on Figure 2 ROIs, radiomic features are then extracted from original and wavelet transformed images of T1, original image of FLAIR and PET. Totally 107 radiomic features can be extracted from each original image, including 14 shape features, 18 first order features, 24 gray level co-occurrence matrix (GLCM) features, 16 gray level run length matrix (GLRLM) features, 16 gray level size zone matrix (GLSZM) features, five neighboring gray tone difference matrix (NGTDM) features, 14 gray level dependence matrix (GLDM) features. In wavelet transformed images with eight decompositions, while H stands for high-pass filter, and L stands for low-pass filter: LLL, LLH, LHL, LHH, HLL, HLH, HHL, HHH; and 744 wavelet features were calculated including the first order, GLCM, GLRLM, GLSZM, NGTDM, GLDM features. For each ROI of participant, a total of 1,037 features were combined from T1, FLAIR, and PET. The radiomics feature extraction was processed using an open-source software named PyRadiomics (Van Griethuysen et al., 2017).

#### 2.2.3 Asymmetric features

The asymmetric features was obtained by calculating the features of the left and right hemispheres, the calculation is according to the following formula:


(1) $$\begin{array}{l} asymmetry=2\times \left(f_{left}-f_{right}\right)/\left(f_{left}+f_{right}\right). \end{array}$$

where *f*_*left*_ and *f*_*right*_ represent the features of the left and right hemispheres, we denote *asymmetry* as the asymmetric feature of the left hemisphere, and −*asymmetry* as the asymmetric feature of the right hemisphere.

#### 2.2.4 Feature selection and model performance measurement

In every participant, these features underwent two normalization procedures. (1) For patients, features were normalized using intra-subject *z*-scoring. For controls, features were normalized using intra-controls *z*-scoring. (2) The patients from (1) were *z*-scored by the mean and standard deviation in the population of controls. The dataset underwent feature selection using Random Forest Classifier, followed by classification using an MLP model. Based on the Scikit-learn module in Python (Abraham et al., 2014), we employed a Random Forest Classifier for feature selection. We conducted five-fold cross-validation on the primary cohort to determine the optimal threshold, which ranged from 0 to the maximum feature importance value. Subsequently, we utilized this selected optimal threshold for feature selection on both the primary cohort and the independent site. Based on the groups of radiomic and morphological experiments, the detailed number of input features after feature selection for each detection analysis task on the frontal lobe and its subregions can be found in Supplementary Table S1.

To determine the optimal data processing and network parameters, we conducted a series of experiments using five-fold cross-validation on the primary cohort. The selection of hyperparameters was based on the performance metrics of each five-fold cross-validation model on their respective validation sets. The MLP model (Spitzer et al., 2022) had two hidden layers containing 40 and 10 nodes, two output nodes, and used a dropout of 0.4 on the input layer for learning more robust representations. We utilized the Adam optimization algorithm and employed the cross-entropy loss function. For the full list of optimized parameters, please refer to Supplementary Table S2. The classification performance was assessed using accuracy, balanced accuracy, specificity, sensitivity, and area under the curve (AUC). Additionally, the detection performance of the lesions in the frontal subregions were evaluated by measuring the overlap between the predicted region and the ground truth label.

In the lateralization detection task for the frontal lobe, data labels are determined based on the surgical side. For a single-sided frontal lobe, label 0 represents a healthy frontal lobe, and label 1 represents a frontal lobe lesion. In the subregion detection task, data labels are determined based on a combination of manual lesion labeling and whether there is overlap with the subregion. For subregions, label 0 represents a healthy subregion, and label 1 represents a subregion lesion. Accuracy refers to the sum of correctly identified healthy and lesion labels divided by the total sum of all labels. Sensitivity is the sum of correctly identified lesion labels divided by the total sum of all lesion labels. Specificity is the sum of correctly identified healthy labels divided by the total sum of all healthy labels. Balanced accuracy is the average of sensitivity and specificity. After obtaining prediction results for all subregions of each subject, predicted lesion regions for that patient are merged together, and the overlap with manually labeled lesion regions is calculated to assess the lesion detection task's performance. The metrics used to evaluate detection performance are determined by calculating the area under the receiver operating characteristic curve (AUC). The optimal threshold for the AUC is determined by maximizing the sum of sensitivity and specificity values to predict detection results.

#### 2.2.5 Feature visualization using UMAP

We performed data visualization to demonstrate the contributions of rdiomic and morphological features in distinguishing patients from the control group. For data visualization, we utilized the Uniform Manifold Approximation and Projection (UMAP), which is an effective tool for non-linear mapping of data points' clustering or grouping and their relative proximity (McInnes et al., 2018).

## 3 Experimental setup and results

### 3.1 Participants characteristics

In primary cohort, 37 children with FLE underwent epilepsy surgery and 20 controls (see Table 1). Of the 37 FLE, 28 were MRI positive, including 26 PET positive and two PET negative, nine were MRI negative and PET positive, 16 frontal lobe lesions in the left hemisphere and 21 in the right hemisphere, mean age ± standard deviation (SD) = 5.9 ± 4.5 years. Of the 12 independent site, seven were MRI positive and PET positive, five were MRI negative and PET positive, seven frontal lobe lesions in the left hemisphere and five in the right hemisphere, mean age ± SD = 5.6 ± 4.4 years. Since it is difficult to obtain a healthy control group of MRI and PET in children, we obtained 20 children with TLE as the control group (mean age ± SD = 5.5 ± 3.3 years).

**Table 1:** Subject characteristics of children with FLE and controls.

**Characteristics**	**Primary cohort**		**Independent site**
	**FLE**	**Controls**	**FLE**
Number	37	20	12
Age (years, mean ± SD)	5.9 ± 4.5	5.5 ± 3.3	5.6 ± 4.4
Sex (male:female)	22:15	11:9	10:2
Hemisphere (left:right)	16:21	9:11	7:5
**Pathology**			
	FCD IA (2)	GG (18)	
	FCD IB (5)		FCD IB (2)
	FCD IIA (7)	PXA (1)	FCD IIA (3)
	FCD IIB (10)		FCD IIB (5)
	MCD (13)	DA (1)	MCD (2)
Seizure-free (>1-year)	37	20	12

SD, standard deviation; NA, not applicable; GG, ganglioglioma; PXA, pleomorphic xanthoastrocytoma; DA, diffuse astrocytoma. Pathology was classified based on the ILAE recommendation of the neuropathologic workup of epilepsy surgery brain tissue.

### 3.2 Test design

In this study, there were a total of 10 groups for radiomic and morphological experiments (see Table 2). The radiomics ROIs included GM, WM, GM & WM, and GWM, while the morphological features (MF) encompassed cortical thickness, gray-white matter intendity contrast, mean curvature, sulcal depth, LCD, FLAIR intensity, doughnut method maps, PET SUVR, GM volume and WM volume. Furthermore, asymmetric features from the left and right hemispheres were added to the original radiomic and morphological features. In primary cohort, all experiments across different groups were conducted using five-fold cross-validation. Finally, the independent site was used to validate the model performance.

**Table 2:** Radiomic and morphological test design.

**Test groups**	**GM**	**WM**	**GM = 1.0**	**WM < 1.0**	**MF**	**Asymmetry**
GM	✓					
GM asymmetry	✓					✓
WM		✓				
WM asymmetry		✓				✓
GM & WM	✓	✓				
GM & WM asymmetry	✓	✓				✓
GWM			✓	✓		
GWM asymmetry			✓	✓		✓
MF					✓	
MF asymmetry					✓	✓

Asymmetry means asymmetric feature of the left and right hemispheres. GM, gray matter; WM, white matter; GM & WM, GM and WM combined; GWM, boundary between GM and WM; MF, morphological feature.

**Figure 3:**
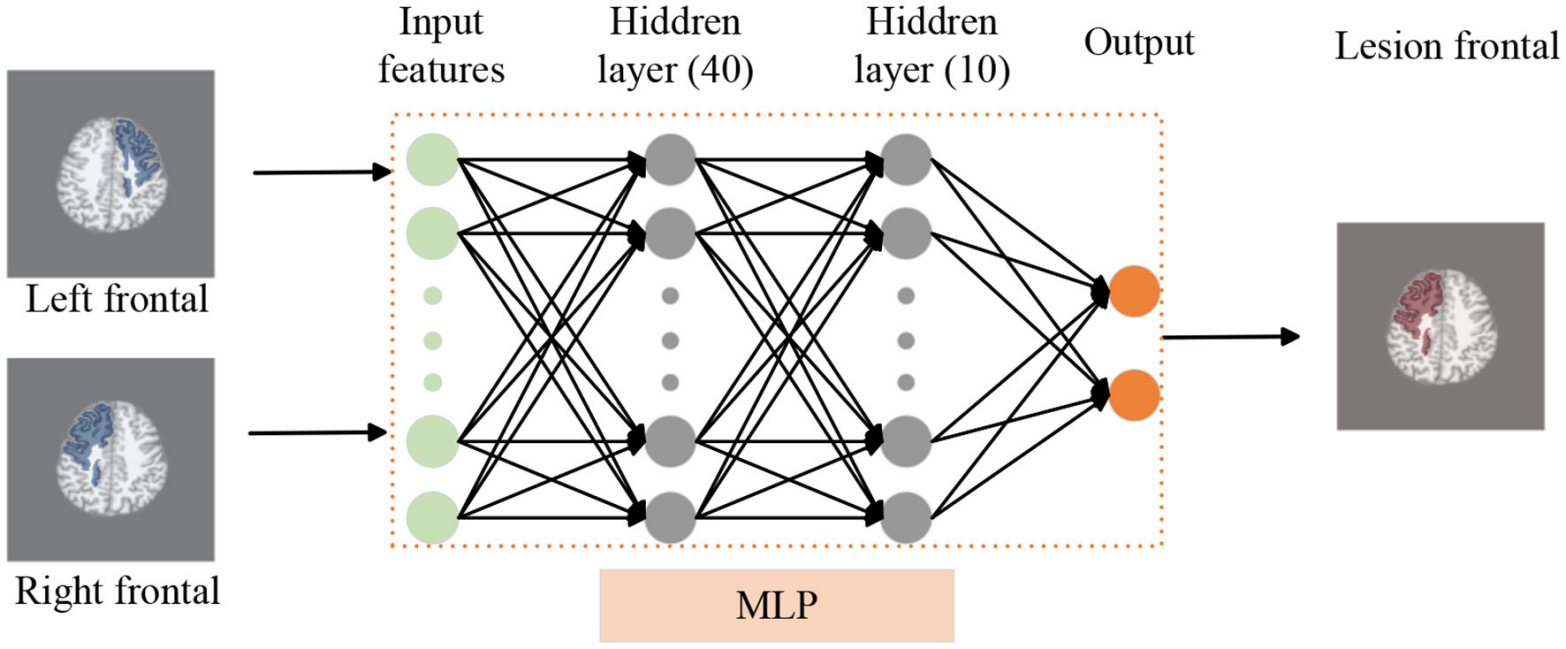
Lobe side detection processing pipeline. We use T1 as background image to display frontal lobe, similar to FLAIR and PET images. We employ an MLP model to detect whether there are lesions in the left and right frontal lobes of patients, thereby identifying the affected frontal lobe with lesions. In this model, the MLP consists of two hidden layers with 40 and 10 nodes respectively.

**Figure 4:**
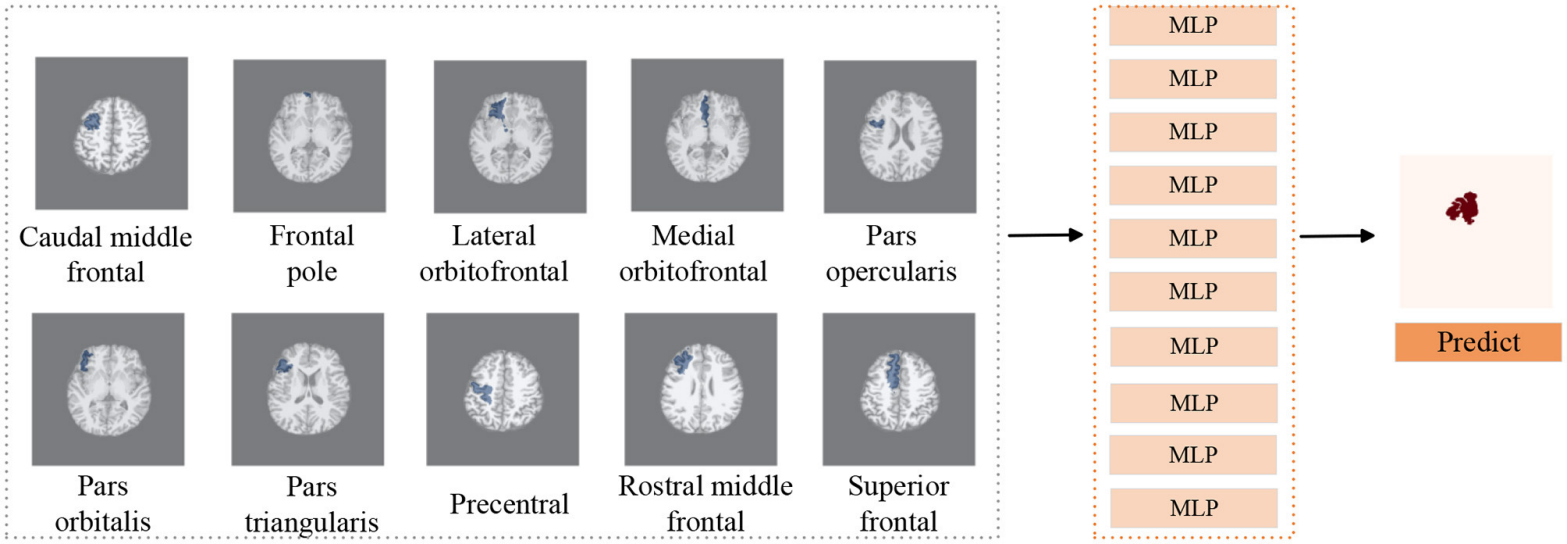
Subregions detection processing pipeline. We use T1 as background image to display subregions, similar to FLAIR and PET images. We utilize MLP classifiers to individually assess the presence of lesions in 10 subregions of the frontal lobe. Subsequently, combining the predictive outcomes of all subregions yields the final prediction of lesion locations.

Our two-stage detection task includes lobe side detection and subregion detection. In the task of detecting FCD in the frontal lobe (see Figure 3), the precise label was determined based on postoperative imaging and surgical side. In the subregion detection task (see Figure 4), previous structural neuroimaging studies have revealed characteristic features of FCD, including cortical thickening, blurring of the GWM, and changes in signal intensity in FLAIR sequences (Spitzer et al., 2022). Moreover, diffusion tensor imaging analysis has demonstrated white matter damage in these patients (Campos et al., 2015), which is confirmed as another major feature of FCD in histopathology (Blumcke et al., 2021). These findings mainly focus on the abnormalities in the GM, WM and GWM regions. Additionally, based on our postoperative imaging of FCD patients, it was observed that the resection area involves both GM and WM. Therefore, for the task of detecting FCD in the subregions, the precise labeling of a subregion determining the overlap between the computed GM & WM regions and the manually annotated labels.

In the training dataset of MLP, we included a total of 37 patients and 20 control subjects. For the detection of lobe sides, each subject encompasses both the left and right frontal lobes, resulting in a total of 114 training datasets. However, in the case of subregion detection, we only considered the subregions of the affected frontal lobes and the single-side frontal lobes of the control group, resulting in a total of 57 training datasets. Instead of traditional morphological feature extraction, a radiomics approach was used to extract a wide range of features, such as shape and texture.

To address the issue of overfitting common in small sample data, as mentioned earlier, we employed a random forest classifier for feature selection. This helped reduce the dimensionality of the data and, consequently, the complexity of the model. The number of selected features after feature selection can be found in Supplementary Table S1, which shows that the filtered features are in the same order of magnitude as the number of samples. Additionally, we utilized five-fold cross-validation to evaluate the performance of the model. Lastly, we introduced Dropout layers in the MLP network to control model complexity and prevent overfitting.

#### 3.2.1 Setup for lobe side detection

For each subject, the brain can be divided into two hemispheres, which can be used as two sets of data for analysis. To detect whether the left and right frontal lobes of patients have FCD (see Figure 3), the left and right hemispheres labels were determined based on postoperative imaging and surgical side. The primary cohort is 114, including 74 patients and 40 controls data. We use five-fold cross-validation to train and test MLP model. The independent site of FLE includes 24 validation data.

#### 3.2.2 Setup for subregions detection

On the basis of determining the frontal lobe of the left and right hemispheres, to further accurately detect the lesion area of the patient's frontal lobe, a classifier was trained separately for each subregion of the frontal lobe (see Figure 4), and the predicted results of all subregions were combined to determine the predicted lesion location of the patient. Considering that the surgical resection area in the postoperative imaging of patients does not include the paracentral region, therefore we did not investigate the paracentral region in the subregions detection task. The manual lesion labels were created based on the postoperative resection areas. The precise labeling of a subregion is determined by calculating whether the subregion overlaps with the manual label.

In the primary cohort, a total of 57 cases were used to evaluate the model performance through five-fold cross-validation. Among patients with FLE, only the affected brain regions were selected, while in the control group, an equal proportion of left or right frontal lobe regions were randomly chosen. The primary cohort of 57 cases consists of 37 patients data and 20 controls data. Similarly, the independent site comprises 12 validation cases.

### 3.3 Performance of radiomics analysis

#### 3.3.1 Primary cohort

In the task of frontal lobe and subregions detection result (see Table 3). For radiomics ROIs with original features, the classification performance AUC of GWM, GM & WM, and WM outperforms that of the GM in lobe side detection (87.2% vs. 83.9% vs. 83.1% vs. 79.4%). In subregions average result, the ROIs of WM and GM & WM had higher AUC than GM and GWM (89.1% vs. 88.2% vs. 88.0% vs. 87.3%). This suggested the radiomics analysis can identify abnormalities in GM, WM, GM & WM, and GWM. When incorporating asymmetric features, all ROIs of radiomics had relative higher accuracy, balanced accuracy, specificity, sensitivity and AUC than the original feature in frontal lobe. However, in the subregion detection result, only the GM and GM & WM asymmetry showed improvement. Overall, both in the frontal lobe and subregions, GM asymmetry had the highest accuracy (93.0% and 85.1%), balanced accuracy (92.0% and 84.4%), specificity (94.8% and 83.3%), sensitivity (89.2% and 85.6%) and AUC (99.0% and 89.9%), suggesting a strong correlation between high-throughput quantitative features and epilepsy.

**Table 3:** Primary cohort radiomics result.

**Step**	**Method**	**ACC (%)**	**BACC (%)**	**SPE (%)**	**SEN (%)**	**AUC (%)**
Frontal	GM	73.7	65.1	89.6	40.5	79.4
	GM asymmetry	**93.0**	**92.0**	**94.8**	**89.2**	**99.0**
	WM	74.6	70.6	81.8	59.5	83.1
	WM asymmetry	92.1	89.9	96.1	83.8	92.9
	GM & WM	74.6	71.3	80.5	62.2	83.9
	GM & WM asymmetry	**93.0**	**92.0**	**94.8**	**89.2**	**98.9**
	GWM	79.8	78.7	81.8	75.7	87.2
	GWM asymmetry	93.9	91.2	98.7	83.8	97.9
Subregions	GM	79.5	78.2	81.2	75.2	88.0
	GM asymmetry	**85.1**	**84.4**	**83.3**	**85.6**	**89.9**
	WM	**84.4**	**83.2**	**84.1**	**82.3**	**89.1**
	WM asymmetry	81.9	80.9	83.1	78.7	88.9
	GM & WM	82.6	81.5	83.6	79.3	88.2
	GM & WM asymmetry	83.2	82.0	82.2	81.8	88.8
	GWM	80.9	80.2	79.9	80.6	87.3
	GWM asymmetry	82.5	81.7	83.4	80.0	86.9

ACC, accuracy; BACC, balanced accuracy; SPE, specificity; SEN, sensitivity; AUC, Area Under the Curve. The best results are respectively marked in bold fonts.

#### 3.3.2 Independent site

The results on the independent site are consistent with the conclusions drawn from the primary cohort in both the frontal lobe and its subregions (see Table 4). In the frontal lobe, the inclusion of asymmetric features from all ROIs improved the classification results, with GM asymmetry and GM & WM asymmetry had the highest accuracy (91.7% and 95.8%), balanced accuracy (91.7% and 95.8%), specificity (91.7% and 100.0%), sensitivity (91.7% and 91.7%) and AUC (99.3% and 99.3%). In subregions, GM asymmetry and WM had the highest sensitivity (79.7% and 78.9%).

**Table 4:** Independent site radiomics result.

**Method**	**Frontal**					**Subregions**
	**ACC (%)**	**BACC (%)**	**SPE (%)**	**SEN (%)**	**AUC (%)**	**SEN (%)**
GM	50.0	50.0	25.0	75.0	53.5	75.2
GM asymmetry	**91.7**	**91.7**	**91.7**	**91.7**	**99.3**	**79.7**
WM	62.5	62.5	75.0	50.0	59.0	**78.9**
WM asymmetry	91.7	91.7	91.7	91.7	91.7	77.6
GM & WM	62.5	62.5	50.0	75.0	59.0	75.1
GM & WM asymmetry	**95.8**	**95.8**	**100.0**	**91.7**	**99.3**	78.4
GWM	54.2	54.2	75.0	33.3	57.3	69.6
GWM asymmetry	87.5	87.5	100.0	75.0	100.0	71.2

The best results are respectively marked in bold fonts.

### 3.4 Performance of morphological analysis

#### 3.4.1 Primary cohort

The performance of morphological analysis results were show in Table 5. After adding asymmetric features in the frontal lobe, there is a higher accuracy (87.7% and 74.6%), balanced accuracy (86.7% and 70.6%), specificity (89.6% vs. 81.8%), sensitivity (83.8% vs. 59.5%) and AUC (95.0% vs. 83.7%) compared to the original features. However, the improvement in AUC (87.9% vs. 86.9%) on the subregions is not significant, indicating that the subregions do not exhibit noticeable morphological abnormalities as observed in the frontal lobe.

**Table 5:** Morphological result.

**Step**	**Method**	**ACC (%)**	**BACC (%)**	**SPE (%)**	**SEN (%)**	**AUC (%)**
**Primary cohort**						
Frontal	MF	74.6	70.6	81.8	59.5	83.7
	MF asymmetry	**87.7**	**86.7**	**89.6**	**83.8**	**95.0**
Subregions	MF	81.6	81.1	78.6	83.5	86.9
	MF asymmetry	82.1	81.0	80.2	81.8	**87.9**
**Independent site**						
Frontal	MF	66.7	66.7	58.3	75.0	69.1
	MF asymmetry	**79.2**	**79.2**	**75.0**	**83.3**	**92.7**
Subregions	MF	NA	NA	NA	63.3	NA
	MF asymmetry	NA	NA	NA	**77.4**	NA

The best results are respectively marked in bold fonts.

#### 3.4.2 Independent site

When adding asymmetric features, whether in the frontal lobe or its subregions, there is an improvement in the classification results on the independent site. In frontal lobe, MF asymmetry had the higher accuracy (79.2%), balanced accuracy (79.2%), specificity (75.0%), sensitivity (83.3%), and AUC (92.7%), while in subregions sensitivity is 77.4%.

### 3.5 Feature visualaization using UMAP on the frontal lobe

**Figure 5:**
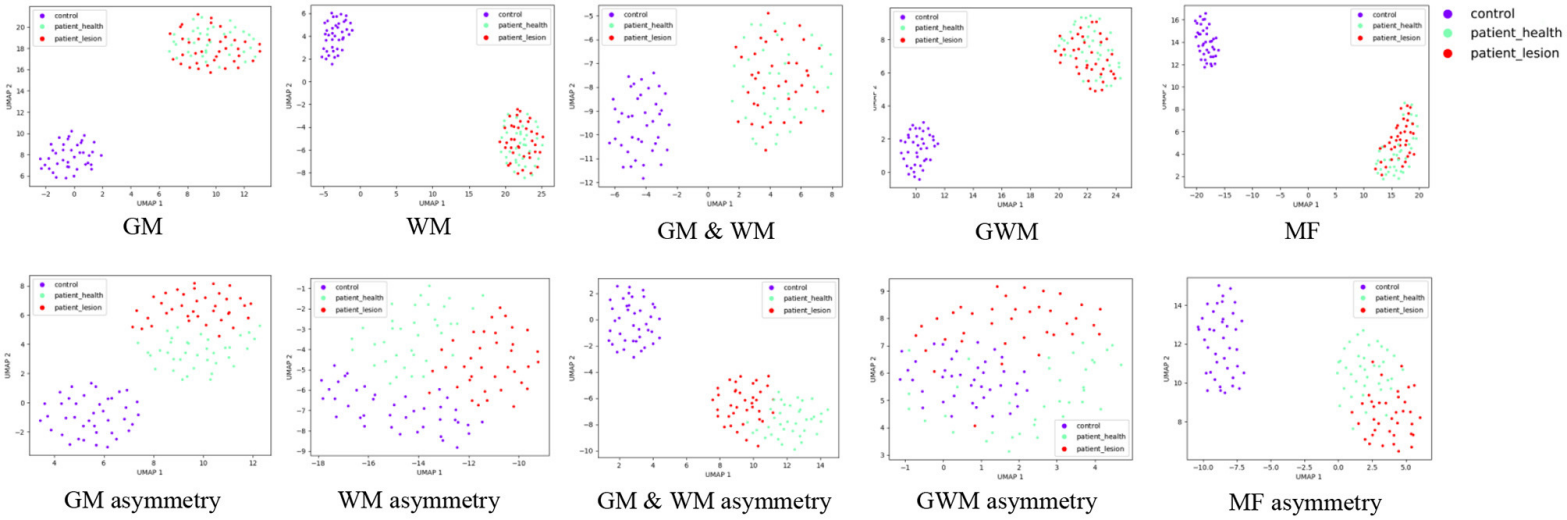
Uniform Manifold Approximation and Projection visualization of features on the frontal lobe, including GM, GM asymmetry, WM, WM asymmetry, GM & WM, GM & WM asymmetry, GWM, GWM asymmetry, MF, MF asymmetry. The red data points represent frontal lobes with lesions, the green points represent healthy frontal lobes, and the purple points represent the control group. MF, morphological feature.

We employed UMAP visualization to gain insights into the effectiveness of features in the frontal lobe detection task. Figure 5 illustrates the UMAP two-dimensional embeddings for GM, GM asymmetry, WM, WM asymmetry, GM & WM, GM & WM asymmetry, GWM, GWM asymmetry, MF, and MF asymmetry features. Visually, for the GM, WM, GM & WM, GWM, and MF, the incorporation of asymmetry features results in a clearer separation among the affected frontal lobe, healthy frontal lobe, and control group, this further validates that the inclusion of asymmetric features can enhance the lobe side detection results.

### 3.6 Performance of two-stage method

In the first-stage lobe side detection task, considering the results of radiomic and morphological methods on both the primary cohort and the independent site, the radiomics method had the higher accuracy (93.0% and 95.8%), balanced accuracy (92.0% and 95.8%), specificity (94.8% and 100.0%), sensitivity (89.2% and 91.7%), and AUC (98.9% and 99.3%) for GM & WM asymmetry, while in the morphological method, the MF asymmetry had the accuracy (87.7% and 79.2%), balanced accuracy (86.7% and 79.2%), specificity (89.6% and 75.0%), sensitivity (83.8% and 83.3%), and AUC (95.0% and 92.7%). From the results, it can be observed that the radiomics method outperforms the morphological method in the lobe side detection task. Therefore, in the first stage, the classification results based on GM & WM asymmetry are utilized as the foundation for subregion detection.

Detailed results of GM & WM asymmetry on the frontal lobe are presented in Table 6. In primary cohort, GM & WM asymmetry detected 33 of 37 FLEs; of the 28 MRI positive frontal lobe cases, 25 were correctly detected; among nine negative cases, eight were detected. The performance is also excellent in an independent site, the detection rate for FLE patients is 91.7%, with 100.0% detection rate for MRI positive cases and 80.0% detection rate for MRI negative patients.

**Table 6:** Frontal detection result with GM & WM asymmetry.

**Dataset**	**FLE (%)**	**MRI positive (%)**	**MRI negtive (%)**
Primary cohort	89.2 (33/37)	89.3 (25/28)	88.9(8/9)
Independent site	91.7 (11/12)	100.0 (7/7)	80.0 (4/5)

**Figure 6:**
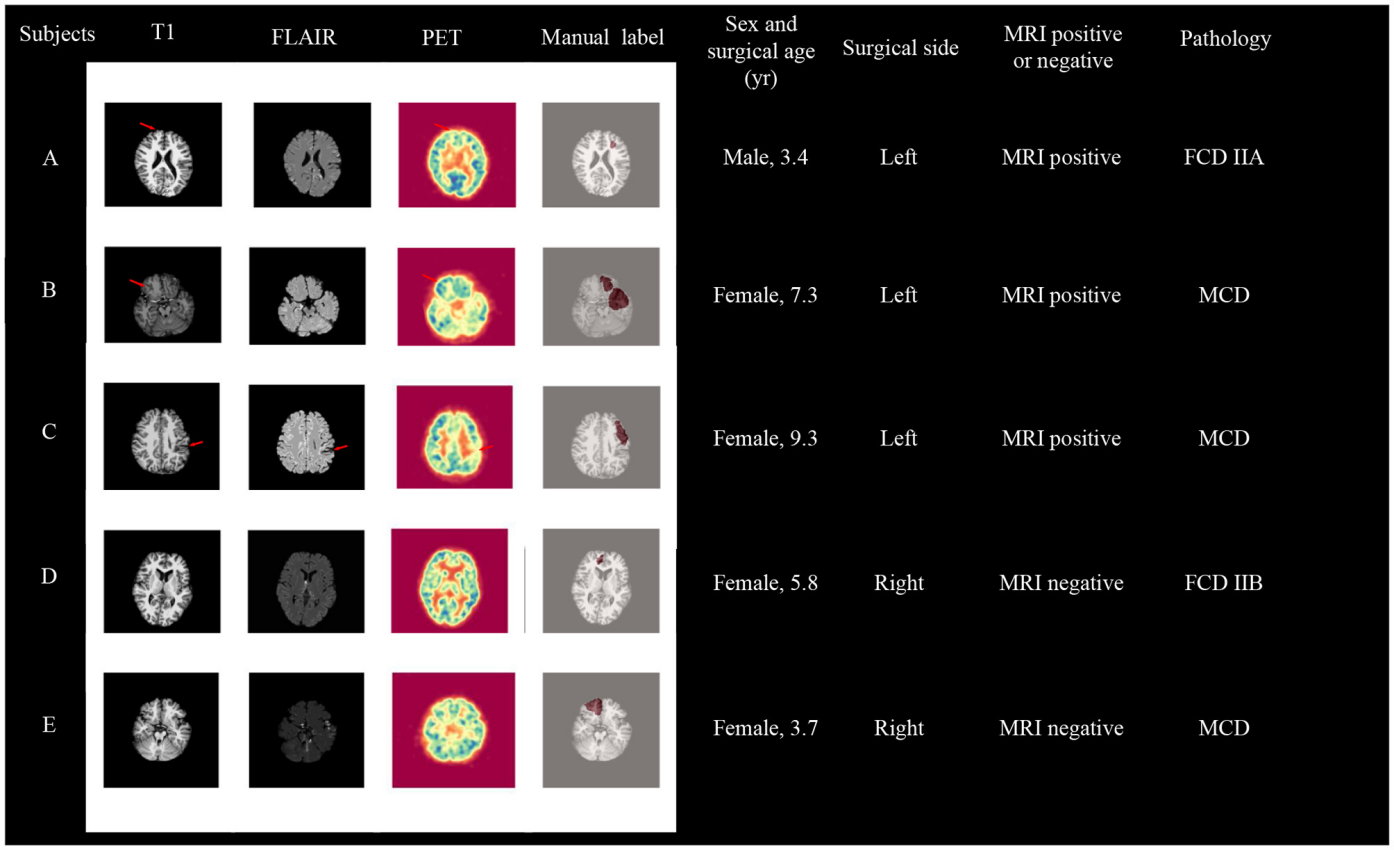
Visualization of patients who failed the first-stage frontal lobe classification, with subject A, B, C, and D coming from the primary cohort, and subject E from an independent site. surgical age is presented in years.

Based on the classification results using GM & WM asymmetry on the frontal lobe, we have presented the information and images of patients classified incorrectly in Figure 6. Among these five subjects, subjects A, B, C, and D are from the primary cohort, and subject E is from an independent site. For these five subjects, the model predicted the affected frontal lobe as a healthy frontal lobe. In detail, for subjects A and B, although they are MRI positive, the imaging abnormalities on the affected frontal lobe on the left side are not prominent. Conversely, there are structural and metabolic abnormalities observed on the contralateral frontal lobe in both T1 and PET scans. For subject C, she represents a unique clinical case. In the clinical assessment before the MRI, she was initially suspected to have brain atrophy caused by a stroke. However, postoperative pathology revealed the presence of MCD, and it cannot be ruled out that this patient may have had FCD coexisting with a stroke. As for subjects D and E, both of them had negative MRI results, and no abnormalities could be identified from the imaging alone. Furthermore, the brain development in pediatric patients is still ongoing, and MRI images are often less clear compared to adult patients, which poses an additional challenge for classification. Moreover, FCD lesions in pediatric patients tend to be larger. Surgical interventions typically focus on resecting the epileptogenic zone rather than all abnormal areas. Considering the limited information available solely from imaging, the determination of the surgical resection area is based on a comprehensive evaluation of anatomical-electrical-clinical information to determine the final surgical approach (Yu et al., 2023).

In the task of subregions detection result, the classification results presented are the average values across all subregions. When incorporating the results from the first-stage lobe side detection task with GM & WM asymmetry (see Table 7). In the radiomics method, both in the primary cohort and the independent site with the highest overlap in GM asymmetry and WM, the primary cohort had the all overlap (55.4% and 53.4%), MRI positive overlap (56.3% and 53.2%) and MRI negative overlap (52.4% and 54.0%). While the independent site had the all overlap (52.4% and 56.8%), MRI positive overlap (48.1% and 52.2%) and MRI negative overlap (59.9% and 64.9%).

**Table 7:** Two-stage detection result.

**Dataset**	**Method**	**Overlap** **(%)**	**MRI** **positive** **(%)**	**MRI** **negtive** **(%)**
**Radiomics**				
Primary cohort	GM	51.1	52.2	47.8
	GM asymmetry	**55.4**	56.3	52.4
	WM	**53.4**	53.2	54.0
	WM asymmetry	50.0	51.2	46.5
Independent site	GM	44.2	45.3	42.4
	GM asymmetry	**52.4**	48.1	59.9
	WM	**56.8**	52.2	64.9
	WM asymmetry	53.4	49.3	60.5
**Morphological**				
Primary cohort	MF	51.7	54.4	43.5
	MF asymmetry	**54.4**	54.5	54.1
Independent site	MF	47.6	41.2	58.7
	MF asymmetry	**53.8**	51.7	57.5

The best results are respectively marked in bold fonts.

In the morphological method, both in the primary cohort and the independent site with the highest overlap in MF asymmetry, the primary cohort had the all overlap (54.4%), MRI positive overlap (54.5%) and MRI negative overlap (54.1%). While the independent site had the all overlap (53.8%), MRI positive overlap (51.7%) and MRI negative overlap (57.5%).

**Figure 7:**
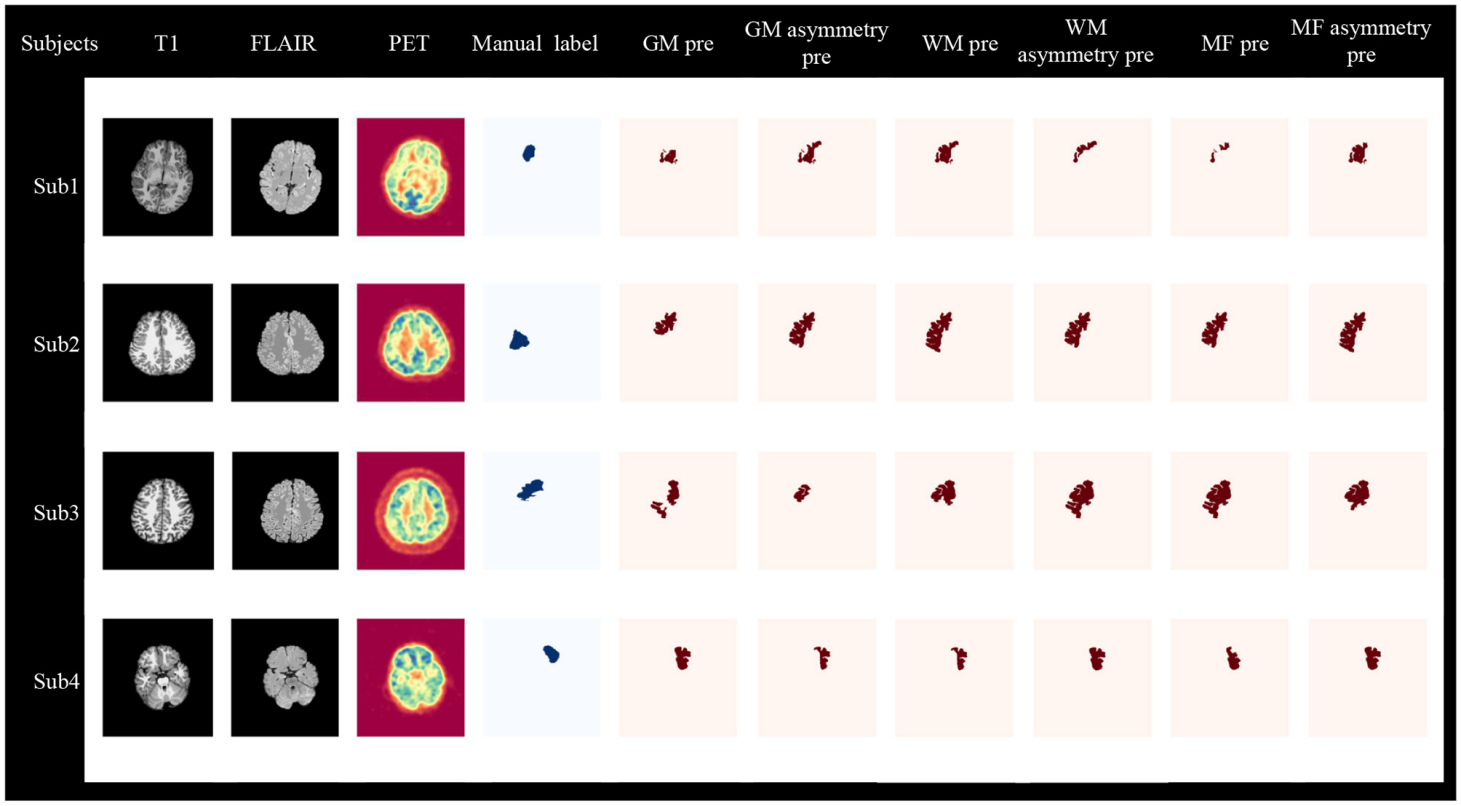
Visualization of two-stage lesion localization results, including GM, GM asymmetry, WM, WM asymmetry, MF, MF asymmetry. Sub1, Sub2, and Sub3 are from the primary cohort, where Sub1 and Sub2 are MRI-positive, and Sub3 is MRI-negative. Sub4 is MRI-positive and comes from an independent site. Sub, subject; pre, predict.

Figure 7 displays four cases from the primary cohort and independent site, demonstrating the diagnostic value of our method. The Sub1 and Sub2 cases originate from the primary cohort, and abnormalities in the lesion area can also be observed from T1, FLAIR, and PET images. GM asymmetry exhibits a higher overlap rate compared to GM, while WM shows a higher overlap rate compared to WM asymmetry, and MF asymmetry demonstrates a higher overlap rate than MF. The Sub3 case is a negative patient with no obvious lesion on the imaging, but our method is still able to detect the affected region using GM asymmetry, WM, MF asymmetry. The sub4 case is a patient from the independent dataset with a positive MRI result. The visualized outcomes and overlap rates remain consistent.

Both the overlap rate results and the visualized outcomes reveal that our proposed two-stage radiomic and morphological combination methods can not only identify positive patients but also effectively detect negative patients. Additionally, to further validate the two-stage detection method, we compared it with the one-stage subregion detection task. Details can be found in Supplementary Table S3. In the primary cohort, the two-stage detection with the radiomics method of GM asymmetry exhibited higher sensitivity (85.6% vs. 56.7%) and overlap (55.4% vs. 43.6%) compared to the one-stage method. Similarly, in the primary cohort with the morphological method, it showed higher sensitivity (81.8% vs. 59.9%) and overlap (54.4% vs. 44.5%) than the one-stage method. These conclusions hold true in the independent dataset as well. Regardless of whether we compare sensitivity or overlap rates, it is evident that the two-stage approach outperforms the one-stage method in FCD lesion detection tasks.

## 4 Discussion

The findings of our study highlight the effectiveness of utilizing an MLP network and radiomics methods for the detection of FCD lesions in multimodal imaging data. We observed that incorporating radiomic and morphological features from different ROIs and asymmetric features from the left and right hemispheres improved the accuracy of FCD detection. Additionally, our approach, which utilized a two-stage detection process based on the clinical diagnostic process of lesion localization, demonstrated excellent performance in detecting FCD lesion.

The proposed asymmetry performs better than basic radiomic and morphological methods in both frontal lobe and subregion diagnosis. Also, it is observed through 2D embedding of UMAP that the accuracy of FCD detection is improved when asymmetric features are added. This indicates the presence of complex structural and functional changes in the ipsilateral and contralateral frontal lobes. This finding is consistent with recent research that views epilepsy as a network-level disorder of the entire brain (van Diessen et al., 2013; Wang et al., 2022). Furthermore, previous studies have also found compensatory mechanisms in the contralateral hemisphere in patients with unilateral FLE (Swartz et al., 1996; Widjaja et al., 2014). The compensatory mechanisms in the contralateral hemisphere to the epileptic focus may further emphasize the significance of asymmetric features, thus enhancing localization accuracy.

Radiologically, the characteristics of FCD on MRI imaging include changes in cortical thickness, blurring of the GWM, gyration anomalies, alterations in T2 or FLAIR signal intensity, and WM signal changes (Colombo et al., 2012; Spitzer et al., 2022). Additionally, while on PET imaging, it is characterized by metabolic abnormalities (Chassoux et al., 2010). The current research focuses on using structural MRI-based methods for identifying FCD characteristics, including surface-based features (Adler et al., 2017), Morphometric Analysis Program (David et al., 2021), and diffusion tensor imaging (Lee et al., 2004; Campos et al., 2015). These methods primarily target abnormalities in the GM, WM, and GWM. In TLE, radiomics methods have shown comparable efficacy to clinically empirical methods (Mo J. et al., 2019), but in FCD, the analysis is limited to extracting texture features from voxels surrounding the MRI lesions, without analyzing abnormalities in GM, WM, and GWM (Kulaseharan et al., 2019). In our study, we applied radiomics methods including original and wavelet transformed images, for ROI analysis of the GM, WM, GM & WM, and GWM in pediatric frontal lobe FCD using both MRI and PET images for the first time. Compared to morphological methods for identifying structural and intensity abnormalities, Radiomics analyzes imaging abnormalities by extracting high-throughput quantitative features from ROIs. After incorporating asymmetry, the results indicate that radiomics methods outperform morphological methods. In lobe side detection, the radiomics methods exhibit an accuracy of 93.0%, balanced accuracy of 92.0%, specificity of 94.8%, sensitivity of 89.2%, and an AUC of 98.9%. In subregion detection, the radiomics methods demonstrate an accuracy of 85.1%, balanced accuracy of 84.4%, specificity of 83.3%, sensitivity of 85.6%, and an AUC of 89.9% in primary cohort. This indicates that radiomic methods provide valuable insights into the detection of FCD lesions and may be helpful in clinical cases where FCD is suspected but no obvious structural abnormalities are present.

In the context of structural and functional abnormalities observed on MRI and PET images, radiomic and morphological methods can identify lesion areas from different perspectives. Our experimental findings demonstrate a significant improvement when combining both methods compared to using morphological methods alone. In the lobe side detection task, the radiomics method outperforms the morphological method. Therefore, we retained the GM & WM detection results in the frontal lobe as the foundation for the combined experiment of radiomic and morphological. The two-stage results demonstrate that after incorporating the morphological information with the first-stage radiomics results, the two-stage radiomics method exhibits similar overlap rates to the morphological method, both in the primary cohort (55.4% and 54.4%) and the independent site (52.4% and 53.8%). This further supports that the radiomics method is as effective as the traditional morphological method in FCD detection.

However, it is important to acknowledge that there are still limitations to consider. Due to the difficulty in obtaining a multimodal healthy control group, we utilized TLE cases as the control group. Furthermore, we faced challenges in benchmarking our two-stage FCD detection method. Finding directly comparable benchmark datasets or conducting rigorous comparative studies is not straightforward, especially in clinical research involving medical data privacy and ethical considerations. These limitations restrict our ability to validate the method's performance on extensive benchmark datasets. Lastly, our research still requires validation on larger multi-center datasets to further assess the method's robustness and generalizability.

In conclusion, our study demonstrates the potential of using radiomics methods for the detection of FCD lesions with GM, WM, GM & WM, and GWM. The asymmetry and integration of radiomic and morphological features, along with the two-stage detection process, improves the efficiency of FCD detection. These findings contribute to the growing body of research on the application of radiomics methods in epilepsy diagnosis. Hence, machine learning utilizing radiomic and morphological features can offer intelligent FCD lesion detection in pre-surgical assessments, aiding in surgical strategizing.

## Data availability statement

The raw data supporting the conclusions of this article will be made available by the authors, without undue reservation.

## Ethics statement

This study has obtained approval from the Ethics Committee of Peking University First Hospital. Written informed consent was provided by the parents of all participants, granting permission for us to use their children's data for research purposes.

## Author contributions

MZ: Writing—original draft, Methodology, Validation. HY: Writing—original draft, Methodology, Validation. GC: Data curation, Writing—review & editing. JH: Data curation, Writing—review & editing. YL: Data curation, Writing—review & editing. JZ: Data curation, Writing—review & editing. NL: Investigation, Writing—review & editing. WZ: Investigation, Writing—review & editing. YC: Investigation, Writing—review & editing. GK: Resources, Supervision, Writing—review & editing. LC: Resources, Supervision, Writing—review & editing.

## References

[B1] Abraham A. Pedregosa F. Eickenberg M. Gervais P. Mueller A. Kossaifi J.. (2014). Machine learning for neuroimaging with scikit-learn. Front. Neuroinform. 8, 14. 10.3389/fninf.2014.00014 24600388 PMC3930868

[B2] Adler S. Wagstyl K. Gunny R. Ronan L. Carmichael D. Cross J. H.. (2017). Novel surface features for automated detection of focal cortical dysplasias in paediatric epilepsy. Neuroimage Clin. 14, 18–27. 10.1016/j.nicl.2016.12.030 28123950 PMC5222951

[B3] Blumcke I. Cendes F. Miyata H. Thom M. Aronica E. Najm I.. (2021). Toward a refined genotype-phenotype classification scheme for the international consensus classification of focal cortical dysplasia. Brain Pathol. 31, e12956. 10.1111/bpa.12956 34196989 PMC8412090

[B4] Bossi Zanetti I. De Martin E. Pascuzzo R. D'Amico N. C. Morlino S. Cane I.. (2023). Development of predictive models for the response of vestibular schwannoma treated with cyberknife^Ⓡ^: a feasibility study based on radiomics and machine learning. J. Pers. Med. 13, 808. 10.3390/jpm13050808 37240978 PMC10221826

[B5] Campos B. M. Coan A. C. Beltramini G. C. Liu M. Yassuda C. L. Ghizoni E.. (2015). White matter abnormalities associate with type and localization of focal epileptogenic lesions. Epilepsia 56, 125–132. 10.1111/epi.12871 25545559

[B6] Chassoux F. Rodrigo S. Semah F. Beuvon F. Landre E. Devaux B.. (2010). Fdg-pet improves surgical outcome in negative MRI Taylor-type focal cortical dysplasias. Neurology 75, 2168–2175. 10.1212/WNL.0b013e31820203a9 21172840

[B7] Cheong E.-N. Park J. E. Jung D. E. Shim W. H. (2021). Extrahippocampal radiomics analysis can potentially identify laterality in patients with MRI-negative temporal lobe epilepsy. Front. Neurol. 12, 706576. 10.3389/fneur.2021.706576 34421804 PMC8372821

[B8] Colombo N. Tassi L. Deleo F. Citterio A. Bramerio M. Mai R.. (2012). Focal cortical dysplasia type IIa and IIb: MRI aspects in 118 cases proven by histopathology. Neuroradiology 54, 1065–1077. 10.1007/s00234-012-1049-1 22695739

[B9] David B. Kröll-Seger J. Schuch F. Wagner J. Wellmer J. Woermann F.. (2021). External validation of automated focal cortical dysplasia detection using morphometric analysis. Epilepsia 62, 1005–1021. 10.1111/epi.16853 33638457

[B10] Desikan R. S. Ségonne F. Fischl B. Quinn B. T. Dickerson B. C. Blacker D.. (2006). An automated labeling system for subdividing the human cerebral cortex on MRI scans into gyral based regions of interest. Neuroimage 31, 968–980. 10.1016/j.neuroimage.2006.01.021 16530430

[B11] Fischl B. (2012). Freesurfer. NeuroImage 62, 774–781. 10.1016/j.neuroimage.2012.01.021 22248573 PMC3685476

[B12] Greve D. N. Salat D. H. Bowen S. L. Izquierdo-Garcia D. Schultz A. P. Catana C.. (2016). Different partial volume correction methods lead to different conclusions: an 18F-FDG-pet study of aging. Neuroimage 132, 334–343. 10.1016/j.neuroimage.2016.02.042 26915497 PMC4851886

[B13] Greve D. N. Svarer C. Fisher P. M. Feng L. Hansen A. E. Baare W.. (2014). Cortical surface-based analysis reduces bias and variance in kinetic modeling of brain pet data. Neuroimage 92, 225–236. 10.1016/j.neuroimage.2013.12.021 24361666 PMC4008670

[B14] Harvey A. S. Cross J. H. Shinnar S. Mathern G. W. Taskforce P. E. S. S. (2008). Defining the spectrum of international practice in pediatric epilepsy surgery patients. Epilepsia 49, 146–155. 10.1111/j.1528-1167.2007.01421.x 18042232

[B15] Huang Y. Chen W. Zhang X. He S. Shao N. Shi H.. (2021). Prediction of tumor shrinkage pattern to neoadjuvant chemotherapy using a multiparametric MRI-based machine learning model in patients with breast cancer. Front. Bioeng. Biotechnol. 9, 662749. 10.3389/fbioe.2021.662749 34295877 PMC8291046

[B16] Huppertz H.-J. Grimm C. Fauser S. Kassubek J. Mader I. Hochmuth A.. (2005). Enhanced visualization of blurred gray-white matter junctions in focal cortical dysplasia by voxel-based 3D MRI analysis. Epilepsy Res. 67, 35–50. 10.1016/j.eplepsyres.2005.07.009 16171974

[B17] Jenkinson M. Beckmann C. F. Behrens T. E. Woolrich M. W. Smith S. M. (2012). FSL. Neuroimage 62, 782–790. 10.1016/j.neuroimage.2011.09.015 21979382

[B18] Kulaseharan S. Aminpour A. Ebrahimi M. Widjaja E. (2019). Identifying lesions in paediatric epilepsy using morphometric and textural analysis of magnetic resonance images. Neuroimage Clin. 21, 101663. 10.1016/j.nicl.2019.101663 30642755 PMC6412079

[B19] Laskowitz D. T. Sperling M. R. French J. A. O'Connor M. J. (1995). The syndrome of frontal lobe epilepsy: characteristics and surgical management. Neurology 45, 780–787. 10.1212/WNL.45.4.780 7723970

[B20] Lawson J. Cook M. Vogrin S. Litewka L. Strong D. Bleasel A.. (2002). Clinical, EEG, and quantitative MRI differences in pediatric frontal and temporal lobe epilepsy. Neurology 58, 723–729. 10.1212/WNL.58.5.723 11889234

[B21] Lee S.-K. Kim D. I. Mori S. Kim J. Kim H. D. Heo K.. (2004). Diffusion tensor MRI visualizes decreased subcortical fiber connectivity in focal cortical dysplasia. Neuroimage 22, 1826–1829. 10.1016/j.neuroimage.2004.04.028 15275939

[B22] Lorio S. Sedlacik J. So P.-W. Parkes H. G. Gunny R. Löbel U.. (2021). Quantitative MRI susceptibility mapping reveals cortical signatures of changes in iron, calcium and zinc in malformations of cortical development in children with drug-resistant epilepsy. NeuroImage 238, 118102. 10.1016/j.neuroimage.2021.118102 34058334 PMC8350142

[B23] McInnes L. Healy J. Melville J. (2018). Umap: Uniform manifold approximation and projection for dimension reduction. arXiv. [preprint]. 10.48550/arXiv.1802.03426

[B24] Mo J. Liu Z. Sun K. Ma Y. Hu W. Zhang C.. (2019). Automated detection of hippocampal sclerosis using clinically empirical and radiomics features. Epilepsia 60, 2519–2529. 10.1111/epi.16392 31769021

[B25] Mo J.-J. Zhang J.-G. Li W.-L. Chen C. Zhou N.-J. Hu W.-H.. (2019). Clinical value of machine learning in the automated detection of focal cortical dysplasia using quantitative multimodal surface-based features. Front. Neurosci. 12, 1008. 10.3389/fnins.2018.01008 30686974 PMC6336916

[B26] Park Y. W. Choi Y. S. Kim S. E. Choi D. Han K. Kim H.. (2020). Radiomics features of hippocampal regions in magnetic resonance imaging can differentiate medial temporal lobe epilepsy patients from healthy controls. Sci. Rep. 10, 19567. 10.1038/s41598-020-76283-z 33177624 PMC7658973

[B27] Rahatli F. K. Sezer T. Has A. C. Agildere A. M. (2020). Evaluation of cortical thickness and brain volume on 3 tesla magnetic resonance imaging in children with frontal lobe epilepsy. Neurol. Sci. 41, 825–833. 10.1007/s10072-019-04135-4 31802343

[B28] Salanova V. Quesney L. Rasmussen T. Andermann F. Olivier A. (1994). Reevaluation of surgical failures and the role of reoperation in 39 patients with frontal lobe epilepsy. Epilepsia 35, 70–80. 10.1111/j.1528-1157.1994.tb02914.x 8112260

[B29] Spitzer H. Ripart M. Whitaker K. D'Arco F. Mankad K. Chen A. A.. (2022). Interpretable surface-based detection of focal cortical dysplasias: a multi-centre epilepsy lesion detection study. Brain 145, 3859–3871. 10.1093/brain/awac224 35953082 PMC9679165

[B30] Swartz B. E. Halgren E. Simpkins F. Fuster J. Mandelkern M. Krisdakumtorn T.. (1996). Primary or working memory in frontal lobe epilepsy: an 18 FDG-pet study of dysfunctional zones. Neurology 46, 737–747. 10.1212/WNL.46.3.737 8618675

[B31] Tan Y.-L. Kim H. Lee S. Tihan T. Ver Hoef L. Mueller S. G.. (2018). Quantitative surface analysis of combined MRI and pet enhances detection of focal cortical dysplasias. Neuroimage 166, 10–18. 10.1016/j.neuroimage.2017.10.065 29097316 PMC5748006

[B32] Téllez-Zenteno J. F. Dhar R. Wiebe S. (2005). Long-term seizure outcomes following epilepsy surgery: a systematic review and meta-analysis. Brain 128, 1188–1198. 10.1093/brain/awh449 15758038

[B33] Téllez-Zenteno J. F. Ronquillo L. H. Moien-Afshari F. Wiebe S. (2010). Surgical outcomes in lesional and non-lesional epilepsy: a systematic review and meta-analysis. Epilepsy Res. 89, 310–318. 10.1016/j.eplepsyres.2010.02.007 20227852

[B34] van Diessen E. Diederen S. J. Braun K. P. Jansen F. E. Stam C. J. (2013). Functional and structural brain networks in epilepsy: what have we learned? Epilepsia 54, 1855–1865. 10.1111/epi.12350 24032627

[B35] Van Griethuysen J. J. Fedorov A. Parmar C. Hosny A. Aucoin N. Narayan V.. (2017). Computational radiomics system to decode the radiographic phenotype. Cancer Res. 77, e104–e107. 10.1158/0008-5472.CAN-17-0339 29092951 PMC5672828

[B36] Wang Z. J. Noh B. H. Kim E. S. Yang D. Yang S. Kim N. Y.. (2022). Brain network analysis of interictal epileptiform discharges from ECOG to identify epileptogenic zone in pediatric patients with epilepsy and focal cortical dysplasia type II: a retrospective study. Front. Neurol. 13, 901633. 10.3389/fneur.2022.901633 35989902 PMC9388828

[B37] Widjaja E. Kis A. Go C. Snead I. I. I. Smith O. C. M. L. (2014). Bilateral white matter abnormality in children with frontal lobe epilepsy. Epilepsy Res. 108, 289–294. 10.1016/j.eplepsyres.2013.12.001 24380759 PMC3951988

[B38] Widjaja E. Mahmoodabadi S. Z. Snead I. I. I. Almehdar O. C. Smith A. M. L. (2011). Widespread cortical thinning in children with frontal lobe epilepsy. Epilepsia 52, 1685–1691. 10.1111/j.1528-1167.2011.03085.x 21627647

[B39] Xu Z. Zhao L. Yin L. Liu Y. Ren Y. Yang G.. (2022). MRI-based machine learning model: a potential modality for predicting cognitive dysfunction in patients with type 2 diabetes mellitus. Front. Bioeng. Biotechnol. 10, 1082794. 10.3389/fbioe.2022.1082794 36483770 PMC9725113

[B40] Yu H. Liu Q. Wang R. Liu C. Sun Y. Wang Y.. (2023). Long-term seizure and developmental outcomes of epilepsy surgery in children under 3 years old: A single-center study of 113 patients. CNS Neurosci. Therap. 1–12. 10.1111/cns.14481 37786975 PMC10805390

[B41] Zhang Q. Liao Y. Wang X. Zhang T. Feng J. Deng J.. (2021). A deep learning framework for 18 F-FDG pet imaging diagnosis in pediatric patients with temporal lobe epilepsy. Eur. J. Nucl. Med. Mol. Imaging 48, 2476–2485. 10.1007/s00259-020-05108-y 33420912 PMC8241642

