# Supplementary material for: Enhanced focal cortical dysplasia detection in pediatric frontal lobe epilepsy with asymmetric radiomic and morphological features
Source: Front Neurosci. 2023 Nov 15;17:1289897. doi: 10.3389/fnins.2023.1289897 (PMC10684781; doi:10.3389/fnins.2023.1289897)
Supplement: Supplementary file 1 [file Data_Sheet_1.PDF]

Supplementary Material

1 SUPPLEMENTARY TABLES AND FIGURES

1.1 Tables

Table S1. The number of features for detection analysis

Region	GM	GM asy	WM	WM asy	GM & WM	GM & WM asy	GWM	GWM asy	MF	MF asy
Frontal	52	37	57	11	29	43	36	30	6	12
Caudal middle frontal	37	34	21	19	24	19	3	22	6	3
Lateral orbitofrontal	12	29	14	7	35	31	20	35	6	17
Medial orbitofrontal	8	37	24	34	22	24	5	32	3	30
Pars opercularis	12	5	6	18	15	20	22	3	6	8
Pars orbitalis	13	34	3	22	10	3	18	21	22	5
Pars triangularis	7	8	19	22	16	7	34	29	12	17
Precentral	22	28	25	25	26	39	27	9	9	24
Rostral middle frontal	2	24	7	21	18	6	17	17	11	2
Superior frontal	16	30	8	8	28	8	8	32	11	11
Frontal pole	7	29	19	38	16	34	5	38	8	5

asy, asymmetry. The number of features retained after feature selection, which is also the input feature count for both the frontal lobe and all subregion detection analysis tasks.

Table S2. List of neural network parameters

Network information	Parameters and descriptions
**Input data**	
Number of subjects	Training set: 37 patients and 20 control, independent site: 12 patients.
Number of input feature	The number of features retained after feature selection.
Shuffle each epoch	True
**Neural network architecture**	
Layers	Input features - Dense (40) Dense (10) - Output (2)
Dropout	0.4
**Training**	
Optimization algorithm	Adam, using the Adam with adaptive learning rates to expedite convergence speed.
Learning rate	0.01
Batch size	64
Epochs	100
Number of folds	5, networks trained with 5-fold cross validation

Table S3. One stage of subregions detection result

Method	Primary cohort		Independent site	
	SEN (%)	Overlap (%)	SEN (%)	Overlap (%)
GM	42.9	33.5	40.4	32.8
GM asymmetry	**56.7**	**43.6**	**53.2**	**39.7**
WM	39.8	23.6	35.2	21.1
WM asymmetry	54.4	39.0	49.7	36.1
GM & WM	43.7	30.3	37.9	28.3
GM & WM asymmetry	52.1	39.6	36.0	27.0
GWM	38.6	25.2	34.7	22.1
GWM asymmetry	57.5	42.8	44.0	31.1
MF	34.9	27.8	25.4	20.3
MF asymmetry	**59.9**	**44.5**	**51.0**	**43.8**

The composition of each subregion dataset is as follows: the primary cohort consists of 37 patients and 20 control subjects, totaling 114 data points; each participant provided data for both the left and right subregions. The independent cohort includes 12 patients, comprising a total of 24 data points. To ensure direct comparability with the two-stage method's results, we focused on the number of subregions with lesions and presented sensitivity as a metric. The calculation of overlap rate aims to ensure the comparability of our results with the two-stage detection method. Specifically, we excluded participants who were misclassified during the frontal lobe lateralization detection stage of the two-stage method, and then computed the remaining overlap rate.
